# Characterization of fish‐specific IFNγ‐related binding with a unique receptor complex and signaling through a novel pathway

**DOI:** 10.1002/2211-5463.13769

**Published:** 2024-02-06

**Authors:** Yasuhiro Shibasaki, Takeshi Yabu, Hajime Shiba, Tadaaki Moritomo, Nobuhiro Mano, Teruyuki Nakanishi

**Affiliations:** ^1^ College of Bioresource Sciences Nihon University Fujisawa Japan; ^2^ Department of Food and Nutrition Nitobe Bunka College Nakano Japan; ^3^ Goto Aquaculture Institute Co., Ltd. Sayama Japan

**Keywords:** cytokine receptor, IFNγ‐related, signal transduction, STAT, type II interferon

## Abstract

Unlike mammals, fish express two type II interferons, IFNγ and fish‐specific IFNγ (IFNγ‐related or IFNγrel). We previously reported the presence of two IFNγrel genes, IFNγrel 1 and IFNγrel 2, which exhibit potent antiviral activity in the Ginbuna crucian carp, *Carassius auratus langsdorfii*. We also found that IFNγrel 1 increased allograft rejection; however, the IFNγrel 1 receptor(s) and signaling pathways underlying this process have not yet been elucidated. In this study, we examined the unique signaling mechanism of IFNγrel 1 and its receptors. The phosphorylation and transcriptional activation of STAT6 in response to recombinant Ginbuna IFNγrel 1 (rgIFNγrel 1) was observed in Ginbuna‐derived cells. Binding of rgIFNγrel 1 to Class II cytokine receptor family members (Crfbs), Crfb5 and Crfb17, which are also known as IFNAR1 and IFNGR1‐1, respectively, was detected by flow cytometry. Expression of the IFNγrel 1‐inducible antiviral gene, Isg15, was highest in Crfb5‐ and Crfb17‐overexpressing GTS9 cells. Dimerization of Crfb5 and Crfb17 was detected by chemical crosslinking. The results indicate that IFNγrel 1 activates Stat6 through an interaction with unique pairs of receptors, Crfb5 and Crfb17. Indeed, this cascade is distinct from not only that of IFNγ but also that of known IFNs in other vertebrates. IFNs may be classified by their receptor and signal transduction pathways. Taken together, IFNγrel 1 may be classified as a novel type of IFN family member in vertebrates. Our findings provide important information on interferon gene evolution in bony fish.

## Graphical abstract

Unique signaling mechanisms of fish‐specific isoforms of IFNγ, IFNγrel 1, from the Ginbuna crucian carp have been identified. Monomeric ligand phosphorylates STAT6 and induces transcriptional activation by binding to the unique pair of receptors, Crfb5 (IFNAR1) and Crfb17 (IFNGR1‐1). These suggest IFNγrel 1 may be classified as a novel type of IFN family member in vertebrates.

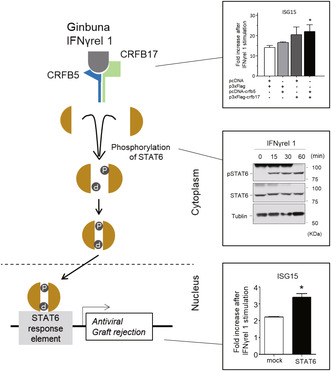

**Abbreviations**
Crfb: cytokine receptor family B
FCM: flow cytometry
IFN: interferon
NLS: nuclear localization signal

Interferon (IFN) is a cytokine responsible for viral interference. In higher vertebrates, IFN family members are classified into three genetically distinct subtypes: types I, II, and III. Each type consists of a distinct ligand conformation, receptor complex and is associated with a JAK–STAT signaling response (reviewed in [1, 2]). Briefly, monomeric type I IFNs (e.g., IFNα, β) bind to the heterodimeric receptors IFNAR1 and IFNAR2. Homodimeric type II IFNs (IFNγ) bind to homodimeric pairs of IFNGR1 and IFNGR2 (tetrameric receptor complex). Monomeric type III IFNλ binds to the heterodimeric receptors, IFNLR1 and IL10R2. Both types I and III predominantly signal through the STAT1‐STAT2 and IRF‐9 complex and induce transcription by interacting with ISRE elements, whereas type II signals through the nuclear translocation of STAT1 homodimers and their interaction with GAS elements. IFNγ cytokines are known as Class II helical cytokines and bind to Class II cytokine receptors (Tables 1 and 2). Genomic analysis in pufferfish and zebrafish revealed the existence of helical Class II cytokine receptor genes, known as cytokine receptor family B (Crfb) [3, 4]. The Crfb genes are functional receptors that are required for the response to ligands [5, 6, 7, 8] (described in Chen *et al*. [8]).

**Table 1:** Currently reported type II interferon receptors and their synonyms.

Species	Receptor name	Synonym	GenBank accession number	References
*Carassius auratus langsdorfii* (Ginbuna crucian carp)	ifngr1‐1	crfb17	AB563726	[24]
	ifngr1‐2	crfb13	AB563727	
*Carassius auratus* (Goldfish)	ifngr1‐1	crfb17	GQ149697	[12]
	ifngr1‐2	crfb13	GQ149698	
*Danio rerio* (Zebrafish)	ifngr1‐1	crfb17	GQ901865	[6]
	ifngr1‐2	crfb13	GQ901864	
	ifngr2	crfb6	EF014956	
*Ctenopharyngodon idella* (Grass carp)	ifngr1‐1	crfb17	AMT92203	[18]
	ifngr1‐2	crfb13	AMT92202	
	ifngr2	crfb6	AMT92201	
*Tetraodon nigroviridis* (Spotted green pufferfish)	ifngr1‐1	crfb17	JF773392	[38]
	ifngr1‐2	crfb13	JF773393	
	ifngr2	crfb6	AJ544909	
*Takifugu rubripes* (Fugu)	ifngr1‐1	crfb17	NM_001360863	[39]
	ifngr1‐2	crfb13	NM_001360834	
	ifngr2	crfb6	NM_001360766	
*Oncorhynchus mykiss* (Rainbow trout)	ifngr1	crfb13	EU244876	[23]
	*ifngr2*	crfb6	EU244877	
*Salmo salar* (Atlantic salmon)	*ifngr2a*	crfb6	NM_001361121	[40]
	*ifngr2b*	crfb6	NM_001361122	
*Acipenser dabryanus* (Dabry's sturgeon)	ifngr1	crfb13	MF741650	[41]
	ifngr2	crfb6	MF741651	
*Arapaima gigas* (Pirarucu)	ifngr1‐1	crfb17	MW349016	[42]
	ifngr2‐1	crfb6‐1	MW349018	
	ifngr1‐2	crfb13	MW349017	
	ifngr2‐2	crfb6‐2	MW349019	
*Siniperca chuatsi* (*Chinese perches*)	ifngr1‐1	crfb17	MH397369	[43]
	ifngr1‐2	crfb13	MH397370	
	ifngr2	crfb6	MH397371	

**Table 2:** Currently reported type II interferons and their receptors.

Species	Ligand	GenBank accession number	Receptor complexes	References
*Carassius auratus langsdorfii* (Ginbuna crucian carp)	IFNγ1	AB570431	ifngr1‐2 + ifngr2	[24]
	IFNγ2	AB570432	ifngr1‐1 + ifngr2	
	IFNγrel 1	AB570433	ifngr1‐1 + ifnar1	This study
*Carassius auratus* (Goldfish)	IFNγ (IFNγ2)	EU909368	ifngr1‐2	[12]
	IFNγrel (IFNγ1)	GQ149696	ifngr1‐1	
*Danio rerio* (Zebrafish)	IFNγ (IFNγ2)	NM_212864	ifngr1‐2 + ifngr2	[6]
	IFNγrel (IFNγ1)	NM_001020793	ifngr1‐1	
*Ctenopharyngodon idella* (Grass carp)	IFNγ	JX196701	ifngr1‐2 + ifngr2	[18]
	IFNγrel	FJ695519	ifngr1‐1 + ifngr1‐2 + ifngr2 ifngr1‐1 + ifngr2 ifngr1‐2 + ifngr2 (Three models are proposed in the report)	
*Labeo rohita* (Rohu)	IFNγrel	KJ874352	Not identified	[44]
*Tetraodon nigroviridis* (Spotted green pufferfish)	IFNγ	KJ524455	ifngr1‐1 + ifngr1‐2	[38]
	IFNγrel	KJ524454	ifngr1‐1 + ifngr1‐2	
*Takifugu rubripes* (Fugu)	IFNγ	AJ616216	Not identified	[45]
*Ictalurus punctatus* (Channel catfish)	IFNγ2a	DQ124250	Not identified	[10]
	IFNγ2b	DQ124251	Not identified	
	IFNγrel (IFNγ1)	DQ124249	Not identified	
*Oncorhynchus mykiss* (Rainbow trout)	IFNγ	AJ616215	ifngr1 *+ ifngr2*	[23]
*Salmo salar* (Atlantic salmon)	IFNγ	AY795563	Not identified	[46]
*Arapaima gigas* (Pirarucu)	IFNγ	MW349022	ifngr1‐2	[42]
	IFNγ‐like	MW349021	ifngr1‐1	
	IFNγrel	MW349020	ifngr1‐1	
*Siniperca chuatsi* (*Chinese perches*)	IFNγ	MH397367	ifngr1‐1 + ifngr1‐2	[43]
	IFNγrel	MH397368	ifngr2	
*Gadus morhua* (Atlantic cod)	IFNγ	FJ356235	Not identified	[47]
*Oreochromis niloticus* (Nile tilapia)	IFNγ	NM_001287402	Not identified	[48]
*Larimichthys crocea* (Large yellow croaker)	IFNγ	KM501500.2	Not identified	[49]
*Epinephelus coioides* (Orange‐spotted grouper)	IFNγ	JX013936	Not identified	[50]
*Dicentrarchus labrax* (European sea bass)	IFNγ	KJ818329	Not identified	[51]
*Lates calcarifer* (Barramundi perch)	IFNγ	NM_001360734	Not identified	[52]
*Acanthopagrus schlegelii* (Black seabream)	IFNγ	KY921614	Not identified	[53]
*Scophthalmus maximus* (Turbot)	IFNγ	KX360748	Not identified	[54]
*Paralichthys olivaceus* (Japanese flounder)	IFNγ	AB435093	Not identified	[55]
*Hippoglossus hippoglossus* (Atlantic halibut)	IFNγ	GU985450	Not identified	[56]

Although their antiviral and pro‐inflammatory functions are homologous to mammalian IFNs, unique IFN systems have been reported in fish. For example, functional intracellular type I IFN derived from alternative splicing was found in teleost fish [9]. Recently, a novel interferon, IFN‐υ, has been identified in zebrafish [8]. Morpholino‐mediated knockdown revealed that Crfb4 and Crfb12 are the functional receptors for IFN‐υ. Because of its unique ligand‐receptor properties, IFN‐υ has been proposed to be a novel type IV IFN. Type II IFNs are also known to have unique features in teleost based on the existence of IFNγrels, which are phylogenetically related to IFNγ found in some teleosts [6, 10, 11, 12, 13, 14] and thought to arise through teleost‐specific tandem duplication of IFNγ gene during evolution [15, 16]. Zaharadnik *et al*. [17] reported two independent groups of IFNγrels occurring in Cypriniformes, Characiformes, and Siluriformes (they called IFNγrel C in their report) and occurring in Acanthomorpha (they called IFNγrel A in their report). A notable structural difference has been reported in Cypriniformes IFNγrel. In grass carp, Zhu *et al*. [18] reported that IFNγrel exists as a homodimer that is connected by two pairs of disulfide bonds. On the other hand, we identified two distinct IFNγrels (IFNγrel 1 and IFNγrel 2) in Ginbuna crucian carp [13] and demonstrated that both exhibit antiviral activity as a monomer despite the fact that IFNγs exist as homodimers.

Several reports have revealed functional differences between IFNγ and IFNγrels [19]. For example, IFNγrel 2 induces a higher antimicrobial response in macrophages than does IFNγ in goldfish [20]. IFNγrel 1, but not IFNγ or IFNγrel 2, increased allograft rejection in Ginbuna crucian carp [21]. Furthermore, a different gene expression profile was induced following IFNγ and IFNγrel stimulation. In particular, in goldfish macrophages, *ceruloplasmin* expression was not affected by IFNγ or in combination with IFNγ and IFNγrel. In contrast, IFNγrel stimulation induced the up‐regulated expression [20]. In pufferfish (*Tetraodon nigroviridis*), expression of *mx* was down‐regulated by IFNγ, whereas its expression was up‐regulated by IFNγrel [14]. These findings suggest that IFNγ and IFNγrel act through different mechanisms.

The intracellular signaling pathway and the receptors for fish IFNγ are similar to that of mammals. Briefly, IFNγ binds to IFNGR1‐1 (Crfb17), IFNGR1‐2 (Crfb13) (teleost IFNGR1 is thought to be duplicated [22]), and IFNGR2 (Crfb6) [23] and activates receptor associated Jak1 and Jak2, which results in STAT1 phosphorylation [6, 20, 24]. However, because IFNγrels are unique cytokines found only in teleost, it is difficult to identify the receptor(s) as mammalian homologs. Therefore, the mechanism of action of IFNγrel remains controversial. In grass carp, three models for homodimeric IFNγrel and its receptor interaction have been proposed (Table 2) [18]. In zebrafish, Aggad *et al*. [6] reported that Crfb 17 (also called IFNGR1‐1) is involved in the IFNγrel 1‐dependent response by morpholino‐mediated knockdown. However, they also discussed the existence of another (paired) receptor (Table 2), which was not identified in a knockdown study. In fact, monomeric IFNs bind to heterodimeric receptors in mammals. Because mammalian IFN receptor genes for monomeric IFNs were identified as heterodimeric [5, 25], heterodimeric receptors for monomeric IFNs appear to be the rule among vertebrates. Therefore, we hypothesized that monomeric IFNγrel 1 interacts with heterodimeric receptor complexes.

We previously reported that IFNγrel 1‐induced antiviral activity is independent of STAT1 phosphorylation or the transcriptional activation of GAS elements in Ginbuna crucian carp [24]. This suggests that monomeric IFNγrel 1 binds to a distinct receptor complex and signals through distinct pathways from that of the IFNγs. Furthermore, our previous findings suggest that the signaling cascade of IFNγrel 1 and IFNγrel 2 are also distinct, because of their characteristic C‐terminal sequence and subcellular localization in target cells: IFNγrel 1 with functional nuclear localization signal (NLS) and IFNγrel 2 without NLS [13]. Grayfer *et al*. [20] reported that STAT1 phosphorylation was observed following IFNγrel 2 stimulation (they called IFNγrel in their report) in goldfish. Functionally, rgIFNγrel 1 administration enhances allograft rejection, which is primarily mediated by cell‐mediated immunity, whereas rgIFNγrel 2 does not [21]. This suggests the presence of distinct signal transduction between IFNγrel 1 and IFNγrel 2.

To test these hypotheses, we examined the receptors and intracellular signaling pathways involved in STAT phosphorylation and signaling. Phosphorylation and transcriptional activation of STAT6 was observed in Ginbuna cell lines. Binding of rgIFNγrel 1 to the Class II cytokine receptor family members, Crfb5 and Crfb17, was detected by flow cytometry (FCM). Dimerization of Crfb5 and Crfb17 was detected by chemical crosslinking. Involvement of STAT6 and its receptors was functionally characterized by expression of the IFNγrel 1‐inducible antiviral gene, *isg15*. These results indicate that IFNγrel 1 interacts with a unique pair of receptors, Crfb5 and Crfb17. Indeed, this cascade is distinct from not only that of IFNγ but also other known IFNs in vertebrates. IFNs may be classified by their receptor and signal transduction pathways. Taken together, IFNγrel 1 may be classified as a novel type in the IFN family in vertebrates. Our findings provide new insight into interferon gene evolution in bony fish.

## Results

### Phosphorylation of STAT6 by following IFNγrel 1 treatment

**Fig. 1:**
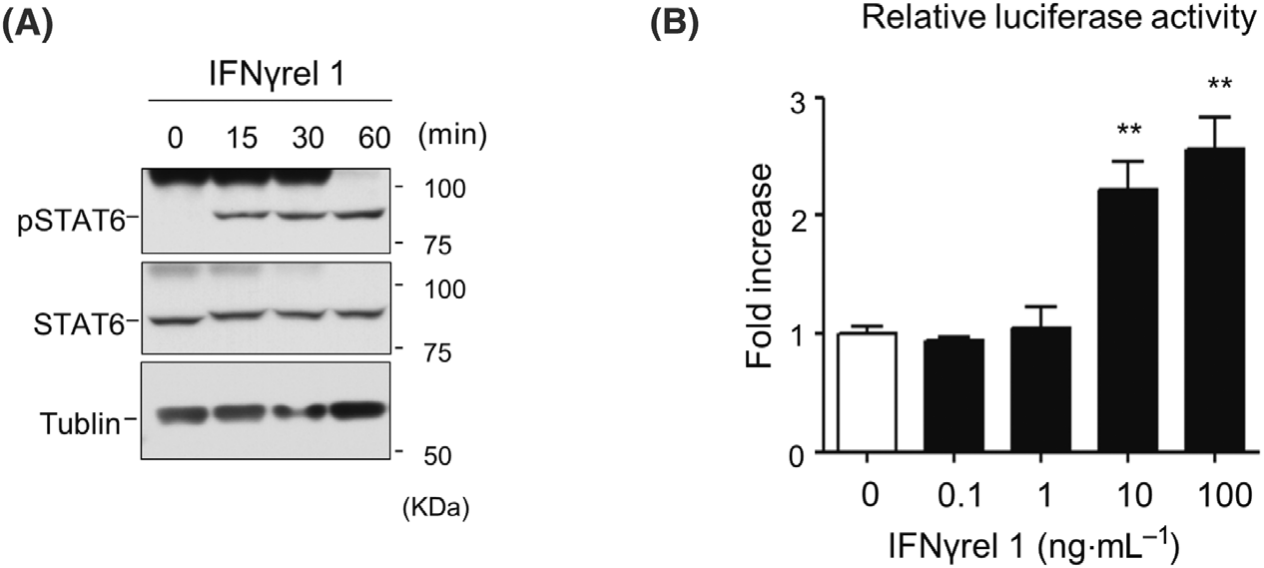
Activation of STAT6 in IFNγrel 1‐stimulated GTS9 cells. (A) GTS9 cells were treated with 100 ng·mL^−1^ of rgIFNγrel 1. Cellular proteins were extracted at the indicated times. The cell lysates were loaded onto an SDS/PAGE gel under reducing conditions. Phosphorylated and total STAT6 protein and tubulin were detected with anti‐STAT6‐Y641, anti‐Stat6, and anti‐tubulin antibodies, respectively, as described in the EXPERIMENTAL PROCEDURES. (B) GTS9 cells were transfected with a construct containing the Cε STAT6 optimal binding element fused to luciferase (pGL4.15‐STAT6). The transfected cells were treated with various concentration of rgIFNγrel 1 for 12 h. Measurement of luciferase activity was done as described in the EXPERIMENTAL PROCEDURES. Each value represents the mean of three independent experiments and error bars represent standard deviations. An asterisk indicates statistical significance using one‐way ANOVA followed by Tukey's *post hoc* test (**P < 0.01).

Based on our previous findings that Ginbuna IFNγrel 1 exists as a monomeric form [13] and does not phosphorylate STAT1 [24], we hypothesized that IFNγrel 1 activates unique signaling pathways. Therefore, we first examined the phosphorylation of STAT proteins in GTS9 cells treated with recombinant Ginbuna IFNγrel 1 (rgIFNγrel 1). As shown in Fig. 1A, phosphorylation of STAT6 was observed, whereas as shown in Fig. S1, the phosphorylation of STAT1, which is phosphorylated by IFN stimulation in known vertebrates [1, 26], was not observed. Phosphorylation of other STATs (STAT2, STAT3, STAT4, and STAT5) was not detected (Fig. S1). Furthermore, neither STAT1 nor STAT6 was phosphorylated following rgIFNγrel 2 stimulation (data not shown).

**Fig. 2:**
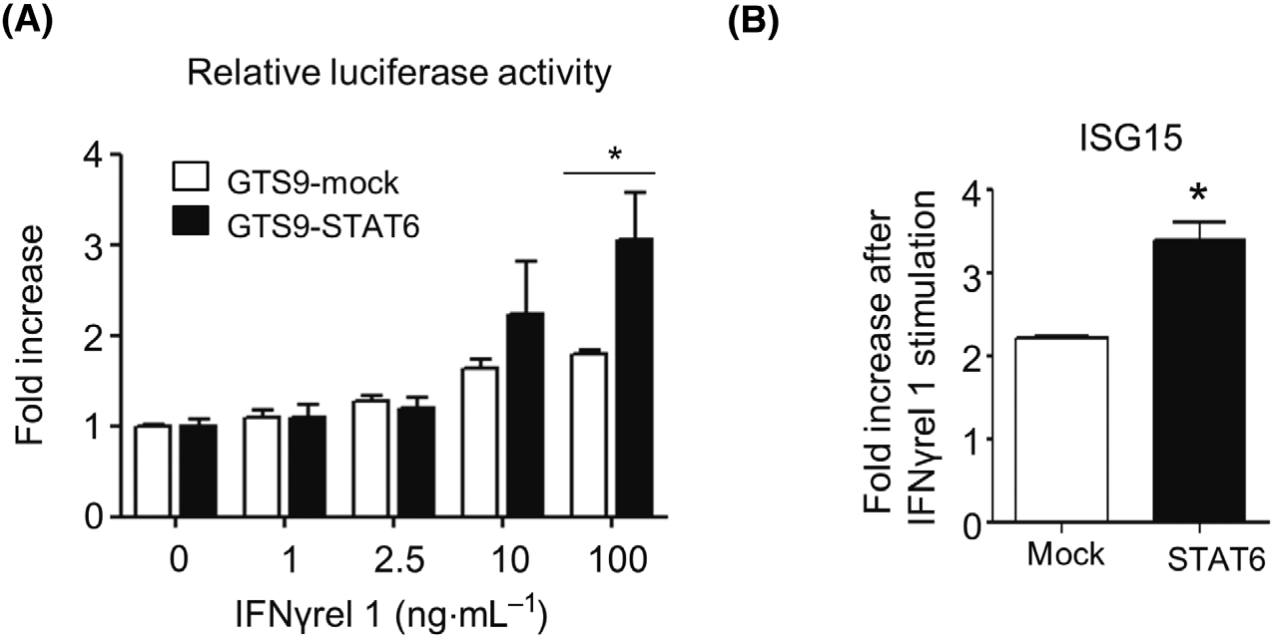
Transcriptional activation in response to Ginbuna crucian carp IFNγrel 1 in GTS9 cells. (A) GTS9 cells were transfected with a FLAG‐STAT6 or mock vector and pGL4.15‐STAT6. Transfected cells were treated with various concentrations of rgIFNγrel 1 for 12 h. Preparation of cell extracts and measurement of luciferase activity were done as described in the EXPERIMENTAL PROCEDURES. Each value represents the mean of three independent experiments and error bars represent standard deviations. (B) GTS9 cells transiently transfected with FLAG‐STAT6 or mock vector were exposed with or without 10 ng·mL^−1^ rgIFNγrel 1 for 6 h. After stimulation, mRNA expression of the IFNγrel 1 inducible gene, *isg15*, was examined by real‐time PCR. The expression levels were normalized to that of the *ef1a* gene and the values were calculated relative to the expression of each transfectant stimulated without rgIFNγrel 1 (mean ± standard error; *n* = 3). An asterisk indicates statistical significance using (A) one‐way ANOVA followed by Tukey's *post hoc* test and (B) a T‐test (*P < 0.05).

A luciferase reporter assay was done to confirm whether IFNγrel 1‐activated STAT6 could induce transcriptional activation. Luciferase activity was induced in a ligand dose‐dependent manner (Fig. 1B). To assess whether the transcriptional activation was attributed to STAT6 phosphorylation, GTS9 cells were transiently transfected with FLAG‐STAT6 plasmid, and luciferase activity in response to rgIFNγrel 1 stimulation was measured. Luciferase activity was significantly increased in STAT6 overexpressing GTS9 cells (Fig. 2A). Moreover, when a dominant‐negative form of STAT6 (Y744F) was transfected into the GTS9 cell line, luciferase activity was significantly attenuated (Fig. S2). We further confirmed the involvement of STAT6 in the IFNγrel 1 signaling. GTS9 cells were transfected with STAT6 and exposed to rgIFNγrel 1. A significant increase in *isg15* mRNA expression, which is known to be induced by IFNγrel 1 stimulation, was observed (Fig. 2B).

### IFNγrel 1 bound to the class II cytokine receptor family members, Crfb5 and Crfb17

**Fig. 3:**
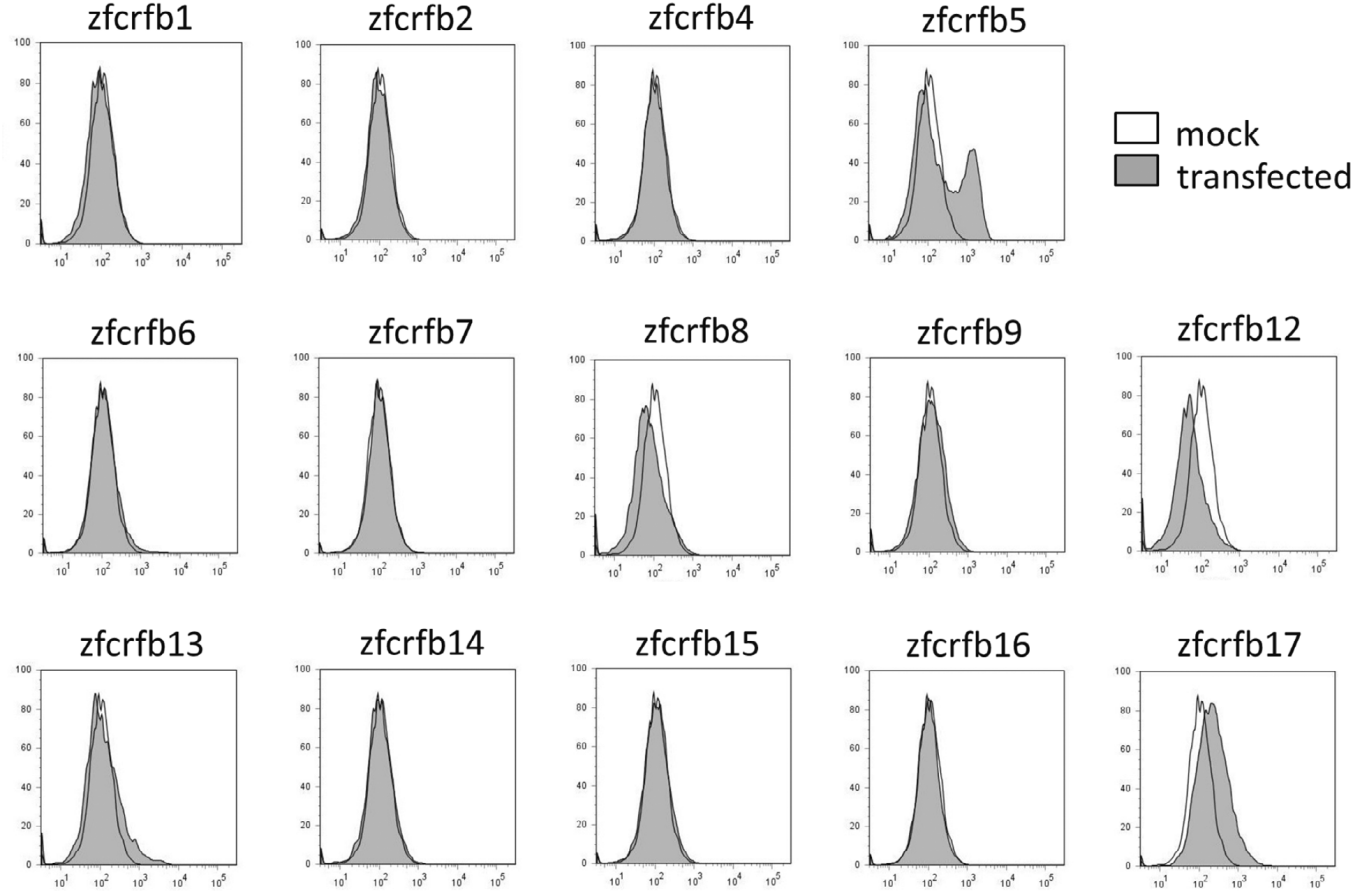
Screening of IFNγrel 1 receptor candidate. HEK293T cells transiently expressing FLAG‐zCrfb1, FLAG‐zCrfb2, FLAG‐zCrfb7, FLAG‐zCrfb8, FLAG‐zCrfb12, FLAG‐zCrfb14, V5‐zCrfb4, V5‐zCrfb5, V5‐zCrfb6, V5‐zCrfb9, V5‐zCrfb13, V5‐zCrfb15, V5‐zCrfb16, or V5‐zCrfb17 were exposed to 100 ng·mL^−1^ of rgIFNγrel 1 for 15 min. Mock vector, pcDNA6/V5‐His A, and p3XFLAG‐CMV™‐14 were used as negative controls. Binding was detected by FCM with an anti‐gIFNγrel 1 antibody.

Recombinant gIFNγrel 1 phosphorylated STAT6 unlike IFNγ in mammals. These results suggest that IFNγrel 1 interacts with receptors other than those that are known for IFNγ. To identify these receptors, we first examined the binding of rgIFNγrel 1 to the zebrafish embryonic cell line, ZE cells, which expresses Class II cytokine receptor family members, Crfb1‐17. Phosphorylation of STAT6 occurred after rgIFNγrel 1 stimulation in ZE cells (Fig. S3) similar to that in GTS9 cells, indicating that rgIFNγrel 1 binds to zebrafish receptors. Candidate receptors for IFNγrel 1, *crfb*s, were isolated from zebrafish (*zcrfb*s) and transiently expressed in HEK293T cells (Fig. S4). Binding of rgIFNγrel 1 to zCrfb5 and zCrfb17, but not to the other zCrfbs, was detected using flow cytometry (Fig. 3).

### Crfb5 and Crfb17 are functional IFNγrel 1 receptors

**Fig. 4:**
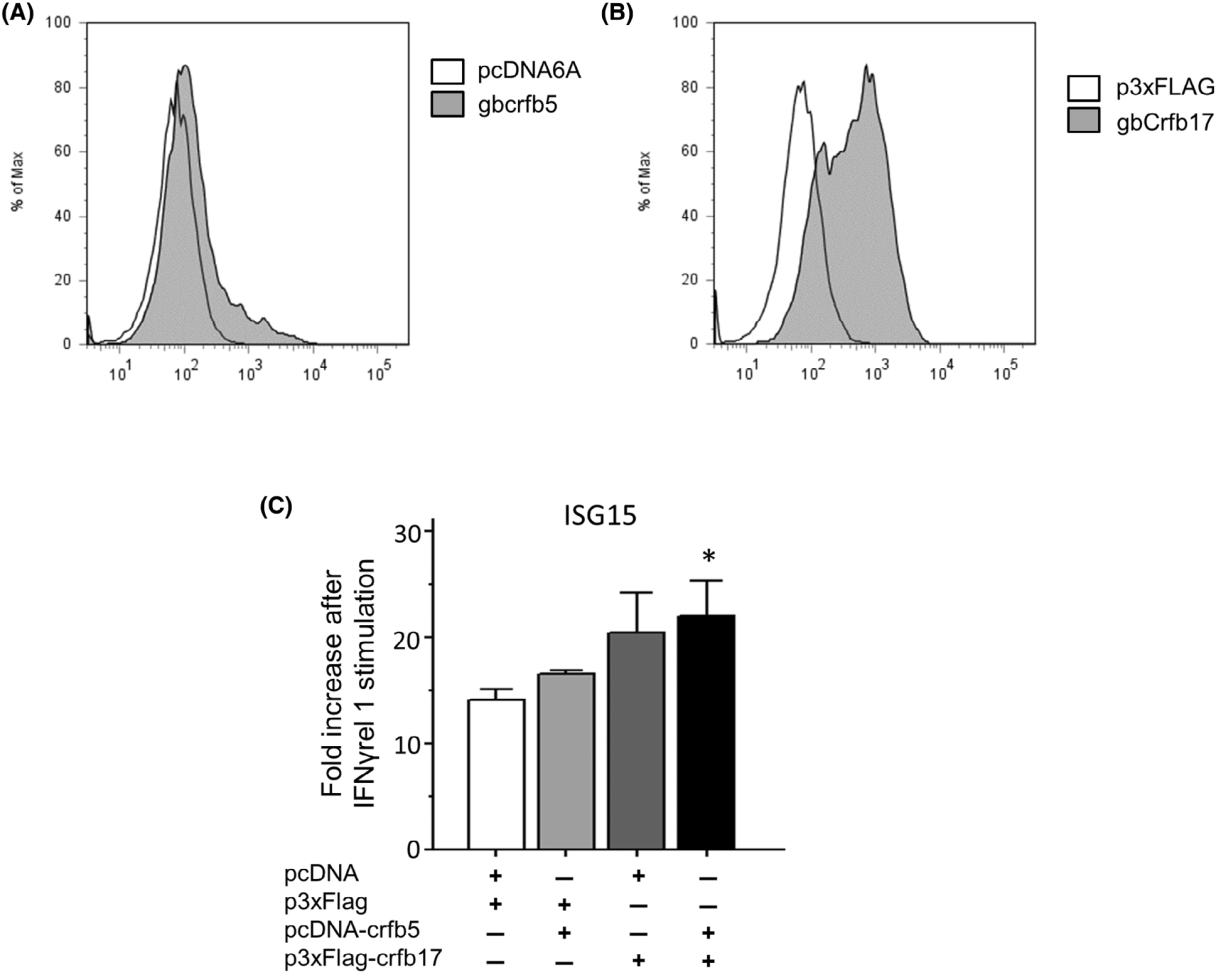
Crfb5 and Crfb17 are functional IFNγrel 1 receptor. HEK293T cells transiently expressing (A) V5‐gCrfb5 or (B) FLAG‐Crfb17 were treated with 100 ng·mL^−1^ of rgIFNγrel 1 for 15 min. Mock vector, pcDNA6/V5‐His A, and p3XFLAG‐CMV™‐14 were used as negative controls. Binding was detected by FCM using an anti‐gIFNγrel 1 antibody. (C) Ginbuna carp‐derived GTS9 cell lines transiently expressing mock vectors, Crfb5, Crfb17, or Crfb5 plus Crfb17 were treated with or without 10 ng·mL^−1^ rgIFNγrel 1 for 6 h. After stimulation, the expression of *isg15* was measured by real‐time PCR. The expression levels were normalized to that of the *Ef1a* gene, and the values were calculated relative to the expression of each transfectant without rgIFNγrel 1 treatment (mean ± standard error; *n* = 4). An asterisk indicates statistical significance using one‐way ANOVA followed by Tukey's *post hoc* test (*P < 0.05).

Since monomeric IFNs are known to bind to heterodimeric receptors [1], we hypothesized that Crfb5 and Crfb17 are heterodimeric receptors for IFNγrel 1. To assess whether crfb5 and crfb17 are functional IFNγrel 1 receptors, we constructed expression vectors, pcDNA6‐gCrfb5 and FLAG‐gCrfb17, and confirmed the binding to gIFNγrel 1 (Fig. 4A,B). Next, we examined the expression of *isg15* in response to rgIFNγrel 1 stimulation in GTS9 cells overexpressing Crfb5 and Crfb17. The highest expression of *isg15* was observed in the GTS9 cells (Fig. 4C).

**Fig. 5:**
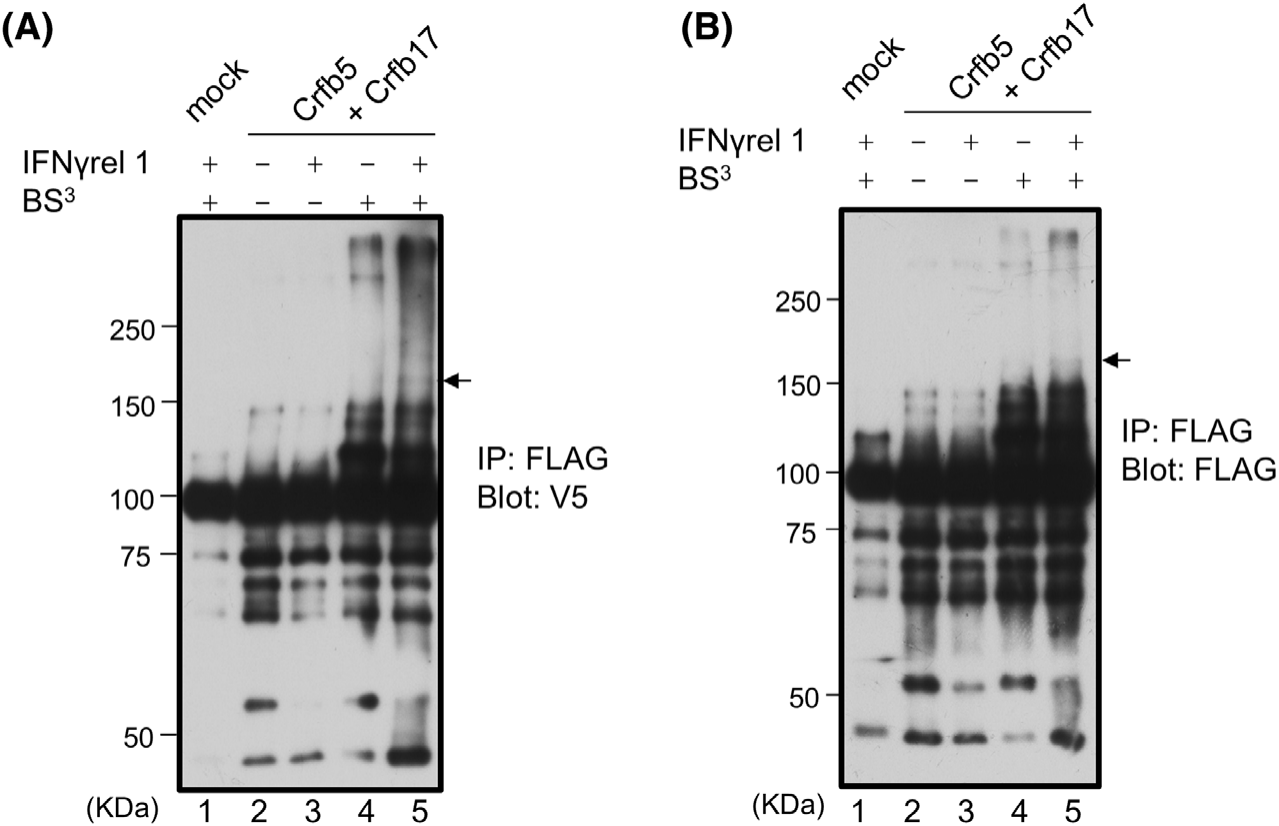
Detection by chemical crosslinking. GTS9 cell lines transiently expressing mock vectors, or Crfb5 and Crfb17, were untreated or treated with 100 ng·mL^−1^ rgIFNγrel 1 for 1 h and then crosslinked with BS^3^. Cell lysates were immunoprecipitated with FLAG antibody and immunoblotted with (A) anti‐V5 antibody or (B) anti‐FLAG antibody. Arrow indicates heterodimeric receptor formation.

Finally, we examined the formation of a receptor complex by chemical crosslinking. pcDNA6‐gCrfb5 and Flag‐gCrfb17 were transiently expressed in GTS9 cells, which were treated with or without rgIFNγrel 1 (approximately 20 kDa) followed by chemical crosslinking. The cell lysates were immunoprecipitated with an anti‐FLAG‐tag antibody, which recognizes 60–70 kDa of Flag‐gCrfb17 and were immunoblotted with an anti‐V5 tag antibody, which recognizes 55–70 kDa of pcDNA6‐Crfb5 (Fig. S5). A band with a molecular mass of 160–170 kDa equivalent to the expected total size of three molecules was detected in IFNγrel 1‐treated cells (Fig. 5A, lane 5). However, the band was attenuated in the absence of IFNγrel 1 (Fig. 5A, lane 4). A band with a similar molecular mass was also detected when blotted with anti‐FLAG antibody (Fig. 5B, lane 5).

## Discussion

Crfb17 is a receptor for IFNγrel 1 as determined by morpholino‐mediated knockdown in zebrafish embryo: [6]. The presence of another receptor, which forms a receptor complex with Crfb17, has been implicated. However, the other receptor that forms a complex with Crfb17 has not been identified thus far. In the present study, FCM‐based receptor screening revealed that IFNγrel 1 binds to Crfb5 and Crfb17 (known as IFNAR1 and IFNGR1‐1 respectively). A chemical crosslinking study also indicated an association between Crfb5 and Crfb17 in response to IFNγrel 1 stimulation. Furthermore, the expression of the IFNγrel 1‐inducible gene, *isg15*, was highest following IFNγrel 1 stimulation when cells over‐expressed pairs of Crfb5 and Crfb17. We previously reported that IFNγrel 1 exists as a monomer, whereas IFNγ occurs as a homodimer. Monomeric Class II cytokines (type I and type III IFNs, as well as IL‐19, IL‐20, IL‐22, and IL‐24) bind to heterodimeric receptors [1, 27, 28]. Taken together, Crfb5 and Crfb17 may be the functional receptors for the IFNγrel 1 ligand.

In the present study, we observed phosphorylation of STAT6 in IFNγrel 1‐stimulated cells, although interferons are not considered to preferentially activate STAT6 in vertebrates (e.g., activation of STAT6 by IFNs has not been reported in vertebrates to date). Furthermore, the antiviral gene, *isg15*, which is important for interferon‐inducible anti‐virus activity [29, 30], was up‐regulated in STAT6 overexpressing cells and attenuated in dominant‐negative STAT6 expressing cells in response to IFNγrel 1. Surprisingly, IFNγrel 1 did not induce phosphorylation of STAT1, which is a predominant signal transducer of IFNs in vertebrates [1, 26]. We previously reported that IFNγ‐induced transcriptional activation occurred through phosphorylation of STAT1 and binding to GAS elements in GTS9 cells [24]. This implies that GTS9 cells express sufficient STAT1. In the present study, we showed that Crfb5 and Crfb17 interact with IFNγrel 1 as a heterodimeric receptor. Although the interaction between IFNγ and IFNGR did not appear to induce STAT6 phosphorylation, IFNAR1 is known to activate not only STAT1, STAT2, and STAT3 but also STAT6 in certain cell types in response to IFNα stimulation in mammals [31, 32]. Therefore, IFNγrel 1 may induce GTS9 cells into an antiviral state through the STAT6 signaling pathway. STAT6 recognizes GAS element in mammals [26, 33, 34]. In our previous study, however, IFNγrel 1 did not induce the transcriptional activation of GAS elements in GTS9 cells [24]. The promoter element recognized by STAT6 is not yet known and further study is needed.

We previously reported the existence of an additional IFNγrel, IFNγrel 2 [13]; however, we have not identified the receptors for IFNγrel 2 thus far. In goldfish, Grayfer *et al*. [12] reported that IFNγrel 2 (which they named IFNγ1 in the paper) binds to IFNGR1‐1 in an *in vitro* crosslinking assay, whereas IFNγ1 (IFNγ2 in the paper) did not bind to IFNGR1‐1, but did bind to IFNGR1‐2. We previously demonstrated that both IFNGR1‐1 and IFNGR1‐2 induce transcriptional activation of GAS elements through STAT1 in response to IFNγ2 and IFNγ1, respectively [24]. Grayfer *et al*. [12] found that IFNγrel 2 activated a different signaling pathway from IFNγ1. Besides, we previously reported that IFNγrel 2 exists as a monomer, and as mentioned in the Introduction, monomeric IFNs bind to heterodimeric receptors [5, 25]. These findings suggest that IFNγrel 2 interacts with another receptor, which forms a heterodimeric receptor associated with IFNGR1‐1. In the present study, however, IFNγrel 2 did not phosphorylate STAT6, which suggests that the receptor for IFNγrel 2 is different from Crfb5. We are currently searching for the receptor for IFNγrel 2 along with its intracellular signaling molecules.

**Fig. 6:**
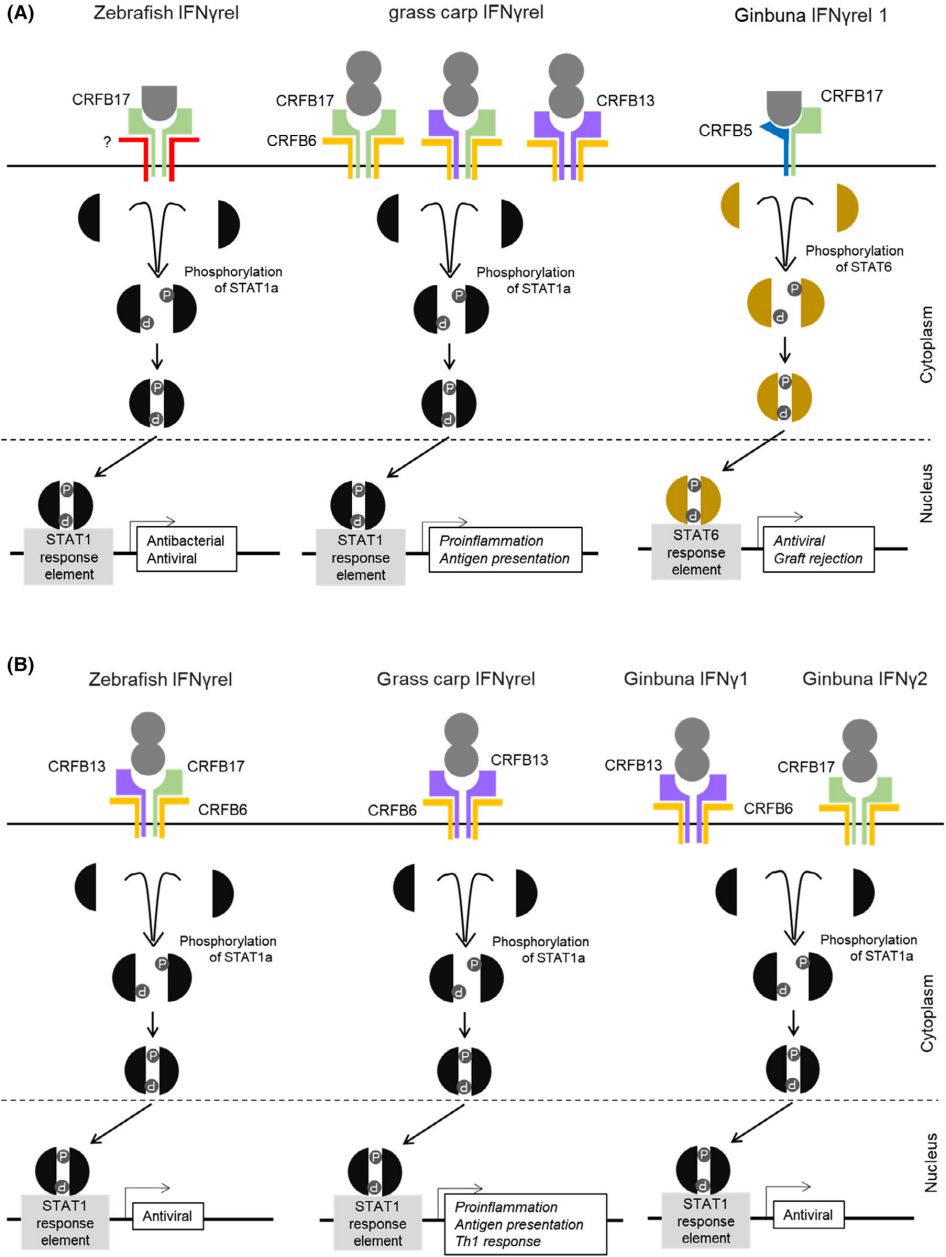
Schematic model for the IFNγrel and IFNγ signaling. Model for the (A) IFNγrel and (B) IFNγ signaling in zebrafish, grass carp and Ginbuna crucian carp.

In summary, IFNγrel 1 phosphorylates STAT6 and induces transcriptional activation by binding to the unique pair of Class II cytokine receptor family members, Crfb5 and Crfb17 (Fig. 6). Indeed, this pathway is distinct from not only that of IFNγ but also those of known IFNs in vertebrates. IFNs may be classified by their receptors and signal transduction pathways [29]. Taken together, IFNγrel 1 is considered a novel type of IFN in vertebrates. Our findings provide new insight into interferon gene evolution in bony fish.

## Materials and methods

### Cell culture

The thymus‐derived GTS9 cell line (developed in our laboratory) from Ginbuna crucian carp (*Carassius auratus langsdorfii*) was cultured in Leibovitz's L‐15 medium (Invitrogen, Carlsbad, CA, USA) supplemented with 10% FBS (Sigma‐Aldrich, St. Louis, MO, USA) at 25 °C. HEK293T cells were obtained from the Health Science Research Resources Bank (Osaka, Japan). The cells were cultured in RPMI medium supplemented with 10% FBS at 37 °C.

### cDNA cloning of Ginbuna crucian carp Crfb5 and stat6 genes

Total RNA from Ginbuna crucian carp and zebrafish splenocytes was extracted using TRIzol reagent (Invitrogen) according to the manufacturer's instructions. Complementary DNA was synthesized using the FirstChoice RLM‐RACE Kit (Life Technologies, Carlsbad, CA, USA) according to the manufacturer's protocol. To obtain partial sequences of the *gcrfb5* (GenBank Accession Number LC752696) and *gstat6* (GenBank Accession Number LC752697) cDNAs, PCR was done using the primers listed in Table S1 in a 40 μL reaction mixture using PrimeSTAR HS DNA Polymerase (Takara, Osaka, Japan). The amplified DNA was subcloned into the pGEM‐T Easy plasmid vector using the TA‐Cloning method (Promega, Madison, WI, USA). Successful cloning of *crfb5* and *stat6* was confirmed by nucleotide sequencing.

### IFNγrel 1 binding assay by flow cytometry

Class II cytokine receptor family members, Crfbs, of zebrafish or Ginbuna crucian carp were subcloned into the p3XFLAG‐CMV™‐14 expression vector (Sigma–Aldrich, St. Louis, MO, USA) or the pcDNA6/V5‐His A vector (Invitrogen). For transient transfection, 2 × 10^5^ HEK293T cells were transfected with 1 μg of the construct DNA using the X‐tremeGENE HP Transfection Reagent (Roche Applied Science, Indianapolis, IN, USA) according to the manufacturer's protocol.

Two days after transfection, HEK293T cells were incubated with 200 ng·mL^−1^ of rIFNγrel 1 for 15 min and washed three times. The cells were then resuspended in PBS containing 0.5% FBS at a concentration of 1 × 10^7^ cells·mL^−1^ and incubated with 1 μg·mL^−1^ of anti‐Ginbuna IFNγrel 1 for 45 min at 4 °C. The cells were then washed three times with PBS containing 0.5% FBS, resuspended, and incubated for 30 min at 4 °C with 1 mL of a 1 : 500 dilution of Alexa 488 goat anti‐rabbit IgG antibody (Life Technologies). The cells were washed an additional three times and then suspended in 0.5 mL of PBS with 2.5 μg·mL^−1^ propidium iodide (Life Technologies). The cells excluding dead cells were analyzed with a FACS Canto (Becton Dickinson).

### Western blot analysis

The cells were lysed in 25 mm Tris–HCl (pH7.4) containing 150 mm NaCl, 0.1% TritonX‐100, and 0.05% SDS. The extracted proteins were resolved by SDS/PAGE and transferred to PVDF membranes (GE Healthcare). Membranes were blocked with StartingBlock (Thermo Fisher Scientific) for 1 h at room temperature and probed with V5 mouse monoclonal antibody (1 : 5000 dilution; Invitrogen, 46–0705), ANTI‐FLAG M2 (1 : 3000 dilution; Sigma, F3165), Anti‐α‐Tubulin (1 : 3000 dilution; Sigma, T9026), Anti‐Actin (1 : 3000 dilution; Sigma, A3853), and anti‐phospho‐STAT1 (Tyr701) (1 : 1000 dilution; Cell Signaling Technology, 9171), anti‐phospho‐STAT2 (Tyr690) (1 : 1000 dilution; Cell Signaling Technology, 4441), anti‐phospho‐STAT3 (Tyr705) (1 : 1000 dilution; Cell Signaling Technology, 9134), anti‐phospho‐STAT4 (Tyr693) (1 : 1000 dilution; Cell Signaling Technology, 5267), anti‐phospho‐STAT5 (Tyr694) (1 : 1000 dilution; Cell Signaling Technology, 9359), anti‐phospho‐STAT6 (Tyr641) (1 : 1000 dilution; Cell Signaling Technology, 9361), and anti‐STAT6 (1 : 1000 dilution; Cell Signaling Technology, 9362) antibodies overnight at 4 °C. The membranes were then washed five times with TBST, incubated with anti‐mouse IgG HRP‐linked (1 : 15 000 dilution; DAKO, P0447) or anti‐rabbit IgG HRP‐linked (1 : 15 000 dilution; Cell Signaling Technology, 7074) secondary antibodies for 1 h at room temperature, and washed five times. The membranes were developed using Western Lightning ECL Pro (Perkin Elmer, Inc., Waltham, MA) and exposed to Hyperfilm ECL (GE healthcare).

### Luciferase reporter assay

The pGL4.15 plasmid, which expresses *Renilla* luciferase, was purchased from Promega (Madison, WI, USA). A nucleotide sequence containing three repeats of the Cε STAT6 optimal binding element (TTCCCAAGAA) [35, 36, 37] was cloned into the pGL4.15 vector (pGL4.15‐STAT6). A pRL‐TK plasmid DNA was used as an internal standard. GTS9 cells were seeded at 2 × 10^4^ cells per well in a 48‐well plate. Then, 300 ng of pGL4.15‐STAT6 and 10 ng of pRL‐TK were co‐transfected into the cells using X‐tremeGENE HP (Roche). After 24 h, the cells were treated with various concentrations of Ginbuna crucian carp IFNγrel 1 for an additional 12 h. The transcriptional activity was examined using a Dual‐Glo Luciferase Assay System (Madison, WI, USA) according to the manufacturer's instructions.

### Expression analysis of isg15 mRNA by real‐time PCR

The synthesis of cDNA was performed using the High‐Capacity cDNA Reverse Transcription Kit (Applied Biosystems, Foster City, CA, USA) according to the manufacturer's instructions. For quantitative real‐time PCR, each target was amplified on the same plate with the housekeeping gene, ef1α, using a Thermal Cycler Dice^®^ Real‐Time System (TaKaRa Bio). The relative mRNA quantities were determined. PCRs were performed with 5 μL of 1 : 100 diluted cDNA, 10 μL of SYBR^®^ Premix Ex Taq (TaKaRa Bio), and 200 nM of each specific primer pair for *isg15* and *ef1α* (Table S1) in a 20 μL mixture. The cycling program was as follows: one cycle at 95 °C for 30 s and 45 cycles at 95 °C for 5 s, followed by 60 °C for 30 s. Raw data were analyzed using the 2^−ΔΔCT^ method relative to *ef1α*, and RQ values were normalized against the non‐sensitized or control group.

### Chemical crosslinking and immunoprecipitation

GTS9 cells transfected with *gcrfb5* and *gcrfb17* or mock vectors were exposed to 100 ng·mL^−1^ of IFNγrel 1 for 1 h at 4 °C and washed three times with ice‐cold PBS buffer. For chemical crosslinking, the cells were suspended in PBS containing 2 mm BS^3^ (Thermo Scientific) and incubated for 30 min at 4 °C, followed by washing three times in Tris‐buffered saline (TBS) (50 mm Tris–HCl, 150 mm NaCl, pH 7.5). The pellets (1 × 10^6^ cells) were resuspended and lysed in 500 μL of 20 mm Tris–HCl (pH 7.5) containing 1% Nonidet P‐40, 1% Triton X‐100, 150 mm NaCl, 5 mm MgCl_2_, 1 mm EDTA, 1 mm EGTA, and a proteinase inhibitor cocktail. Cellular debris was removed by centrifugation at 13,000 *g* for 10 min at 4 °C. The cell lysates were incubated with anti‐FLAG antibody, followed by incubation with protein G Sepharose (GE Healthcare), sedimentation, and five washes with 20 mm Tris–HCl (pH 7.5) containing 1% Nonidet P‐40, 1% Triton X‐100, 150 mm NaCl, 5 mm MgCl_2_, 1 mm EDTA, 1 mm EGTA, and proteinase inhibitor cocktail.

## Conflict of interest

The authors declare no conflict of interest.

### Peer review

The peer review history for this article is available at https://www.webofscience.com/api/gateway/wos/peer‐review/10.1002/2211‐5463.13769.

## Author contributions

YS, TY, and TN conceptualized and designed the study. YS, TY and HS acquired the data. YS, HS, and TM performed data analysis. YS and TN wrote the original draft. TM, NM, and TN supervised the study. All authors reviewed the manuscript.

## Supporting information


**Fig. S1.** Phosphorylation of STAT6 by following IFNγrel 1 treatment in GTS9 cells.
**Fig. S2.** Dominant‐negative form of STAT6 transfected GTS9 cells showed attenuated transcriptional activity.
**Fig. S3.** Phosphorylation of STAT6 by following IFNγrel 1 treatment in zebrafish ZE cells.
**Fig. S4.** Western blot analysis of zebrafish Crfb transfected HEK293T cells.
**Fig. S5.** Western blot analysis of Ginbuna crucian carp Crfb transfected GTS9 cells.

**Table S1.** Oligonucleotide primer sequences.

## Data Availability

The nucleotide sequence data that support the findings in this study are openly available in the GenBank of NCBI at https://www.ncbi.nlm.nih.gov/, accession number [LC752696] and [LC752697].
