# Supplementary material for: Characterization of fish‐specific IFNγ‐related binding with a unique receptor complex and signaling through a novel pathway
Source: FEBS Open Bio. 2024 Feb 6;14(4):532–44. doi: 10.1002/2211-5463.13769 (PMC10988753; doi:10.1002/2211-5463.13769)
Supplement: Supplementary file 1 — Fig. S1. Phosphorylation of STAT6 by following IFNγrel 1 treatment in GTS9 cells. Fig. S2. Dominant‐negative form of STAT6 transfected GTS9 cells showed attenuated transcriptional activity. Fig. S3. Phosphorylation of STAT6 by following IFNγrel 1 treatment in zebrafish ZE cells. Fig. S4. Western blot analysis of zebrafish Crfb transfected HEK293T cells. Fig. S5. Western blot analysis of Ginbuna crucian carp Crfb transfected GTS9 cells. [file FEB4-14-532-s001.pdf]

Fig. S1

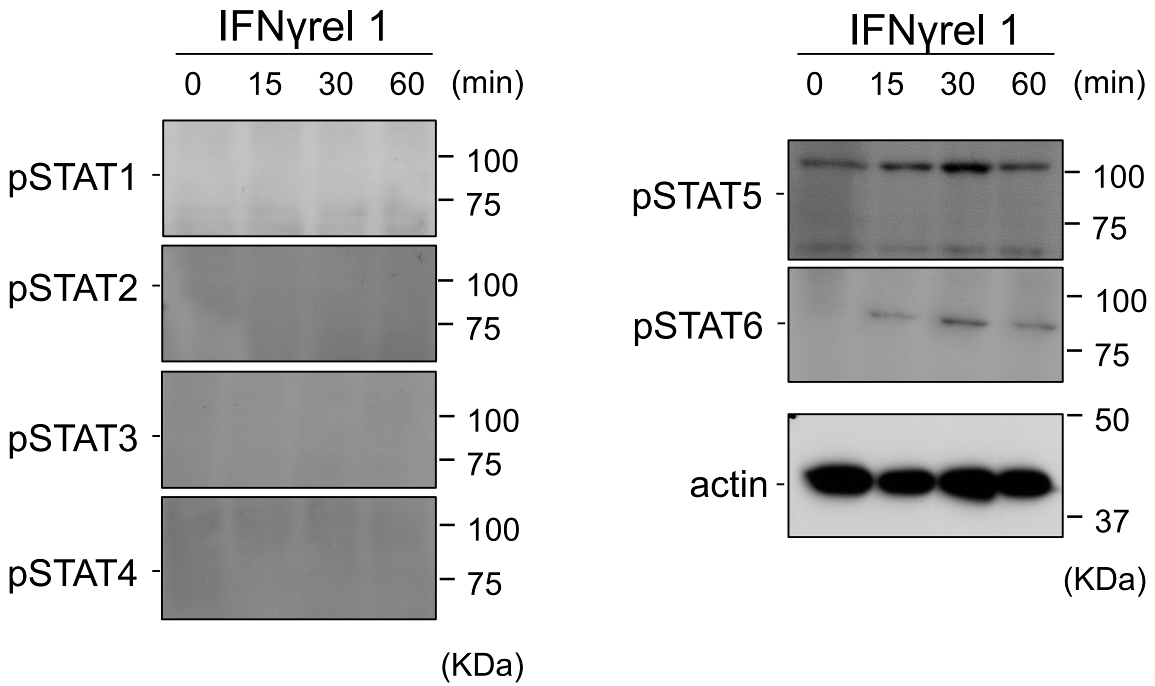

Fig. S1 Phosphorylation of STAT6 by following IFNγrel 1 treatment in GTS9 cells.

GTS9 cells were treated with 100 ng/ml of rgIFNγrel 1. Cellular proteins were extracted at the indicated times. The cell lysates were loaded onto an SDS-PAGE gel under reducing conditions. Phosphorylated STAT proteins and actin were detected as described in the Methods.

Fig. S2

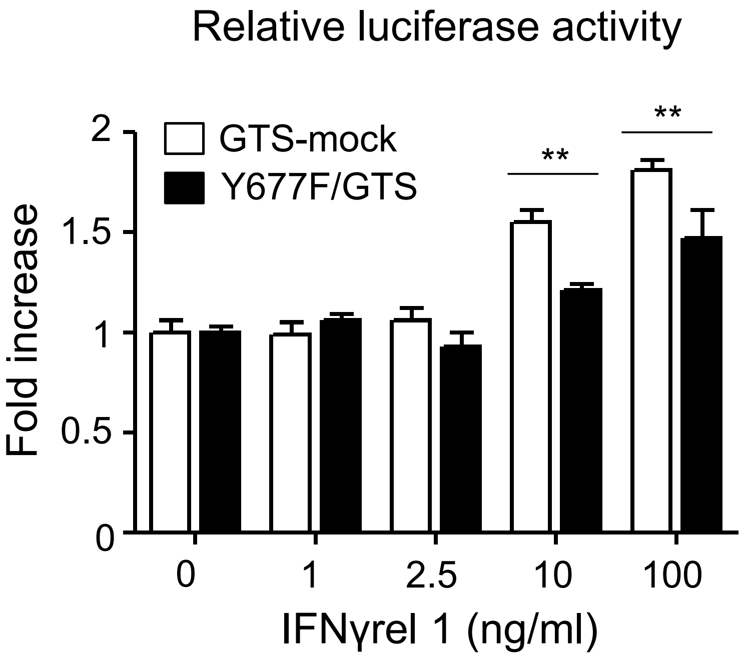

Fig. S2 Dominant-negative form of STAT6 transfected GTS9 cells showed attenuated transcriptional activity.

GTS9 cells stably express the Cε STAT6 optimal binding element fused to luciferase were either transfected with the dominant-negative form of STAT6 (Y744F) or mock vector. The transfected cells were treated with various concentrations of rgIFNγrel 1 for 12 h. Measurement of luciferase activity was done as described in the Methods. Each value represents the mean of three independent experiments, and error bars represent standard deviations. An asterisk indicates statistical significance using two-way ANOVA followed by Bonferroni post hoc test (**: $p < 0.01$).

Fig. S3

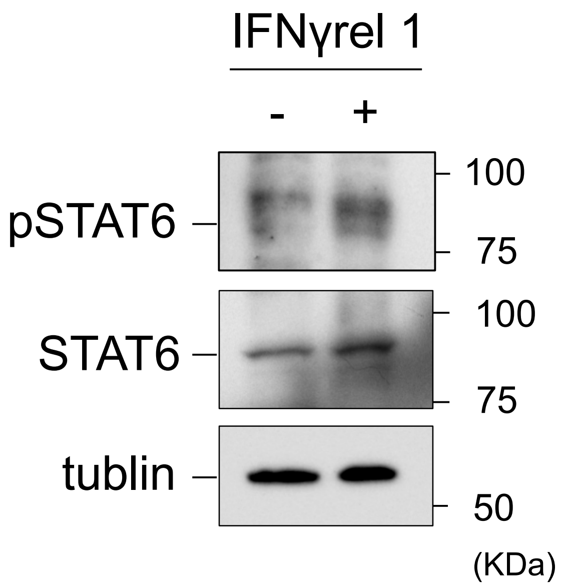

Fig. S3 Phosphorylation of STAT6 by following IFNγrel 1 treatment in zebrafish ZE cells.

Zebrafish embryonic ZE cells were treated with 100 ng/ml of rgIFNγrel 1 and treated for 30 minutes. Cellular proteins were extracted, and the cell lysates were loaded onto an SDS-PAGE gel under reducing conditions. Phosphorylated STAT proteins and actin were detected as described in the Methods.

Fig. S4

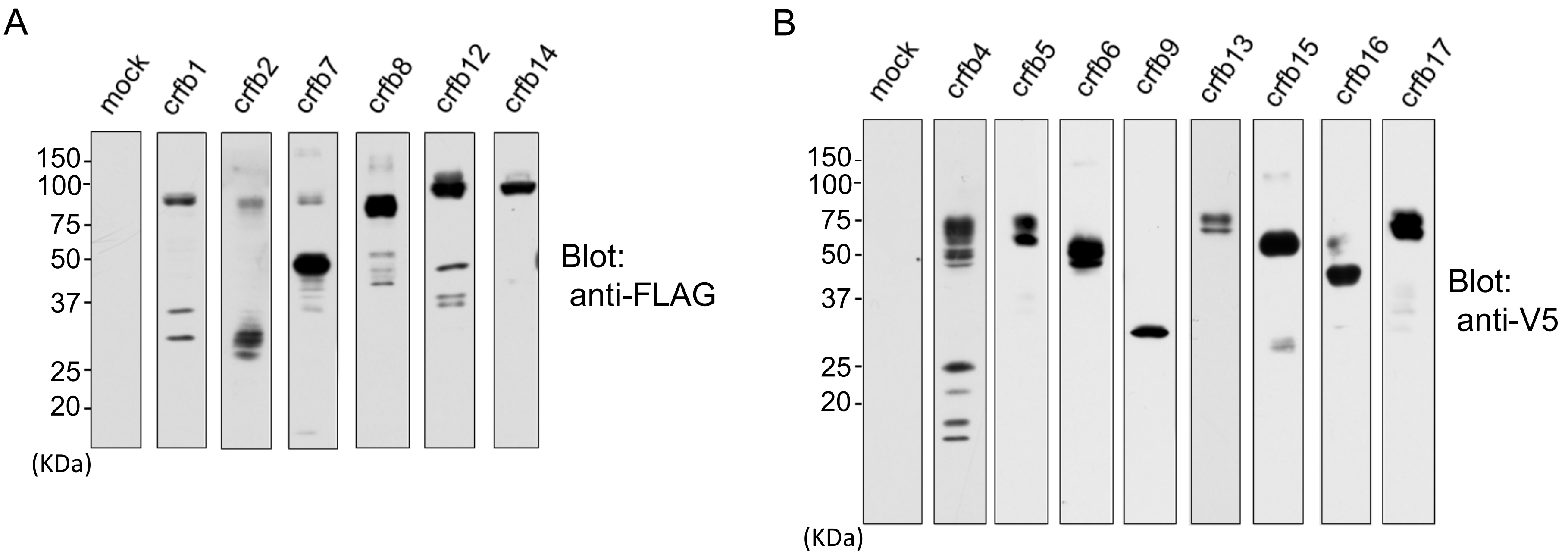

Fig. S4 Western blot analysis of zebrafish Crfb transfected HEK293T cells. HEK293T cells were transiently transfected with FLAG-zCrfb1, FLAG-zCrfb2, FLAG-zCrfb7, FLAG-zCrfb8, FLAG-zCrfb12, FLAG-zCrfb14, V5-zCrfb4, V5-zCrfb5, V5-zCrfb6, V5-zCrfb9, V5-zCrfb13, V5-zCrfb15, V5-zCrfb16, or V5-zCrfb17. Membrane proteins were extracted, and immunoblotted with (A) anti-FLAG antibody or (B) anti-V5 antibody.

Fig. S5

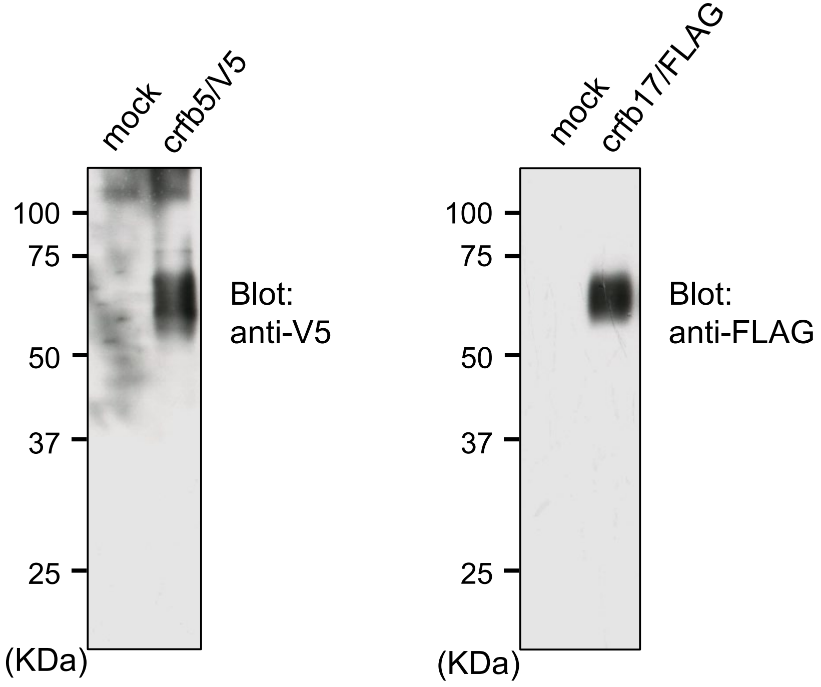

Fig. S5 Western blot analysis of Ginbuna crucian carp Crfb transfected GTS9 cells.

GTS9 cells were transiently transfected with Flag-gCrfb17 and pcDNA6-gCrfb5. Membrane proteins were extracted and immunoblotted with anti-V5 antibody or anti-FLAG antibody.
