# Supplementary material for: Characterization of fish‐specific IFNγ‐related binding with a unique receptor complex and signaling through a novel pathway
Source: FEBS Open Bio. 2024 Feb 6;14(4):532–44. doi: 10.1002/2211-5463.13769 (PMC10988753; doi:10.1002/2211-5463.13769)
Supplement: Supplementary file 2 — Table S1. Oligonucleotide primer sequences. [file FEB4-14-532-s002.docx]

| **Supplementary Table 1. Oligonucleotide primer sequences**. | | | | | | | |
| --- | --- | --- | --- | --- | --- | --- | --- |
| Primer name |  | Sequence (5'→3') |  | Use |  | GenBank accession number |  |
| EF1-α/F | | ACCCCAAGGCTCTCAAATCT | | Expression analysis | | AB491676 | |
| EF1-α/R | | TCAACGCTCTTGATGACACC | |  |  |  |  |
| ISG15 | | TCTGCACTCTGGATCAGTGG | | Expression analysis | | LC752645 | |
| ISG15 | | ACTTCATACGTCCCGGTCTG | |  |  |  |  |
| Zebrafish crfb1_cloning _F | | TGATTAGGACCTGGACATGCTG | | cDNA cloning | | NM_001079681.1 | |
| Zebrafish crfb1_cloning _R | | TAACAAAGCCACTGGCAACTTC | |  |  |  |  |
| Zebrafish crfb2_cloning _F | | TGGATGTCTCAATGAGGGCAG | | cDNA cloning | | NM_001077626.2 | |
| Zebrafish crfb2_cloning _R | | ACTCTGTTCCATCTTCACTGC | |  |  |  |  |
| Zebrafish crfb4_cloning _F | | TTCTCAGCGTGCGCACTTC | | cDNA cloning | | NM_001083868.1 | |
| Zebrafish crfb4_cloning _R | | AATGTGGCTACAGATTGG | |  |  |  |  |
| Zebrafish crfb5_cloning _F | | TTCTCCTCAGTGAGCGAAACCGA | | cDNA cloning | | EF014955.1 | |
| Zebrafish crfb5_cloning _R | | TGTGTGTGAATCCTTCAGTCCTG | |  |  |  |  |
| Zebrafish crfb6_cloning _F | | TCAGCAAGAACGACGCTTTCTC | | cDNA cloning | | AM039829.1 | |
| Zebrafish crfb6_cloning _R | | TGCATTTTTTATGGAGTTCAA | |  |  |  |  |
| Zebrafish crfb7_cloning _F | | GTATCGTAGTCAGAGTCCTTC | | cDNA cloning | | NM_001077625.1 | |
| Zebrafish crfb7_cloning _R | | TCCTGATGATTTCACACTCTTC | |  |  |  |  |
| Zebrafish crfb8_cloning _F | | TGAAAACTTCTCCAGCTTCATG | | cDNA cloning | | NM_001079676.2 | |
| Zebrafish crfb8_cloning _R | | TCCTTCATTATATCGCCTCATTCG | |  |  |  |  |
| Zebrafish crfb9_cloning _F | | TGGCCTACTGGATGCCTTTTAAC | | cDNA cloning | | NM_001045279.1 | |
| Zebrafish crfb9_cloning _R | | GAGCTGAGTTTGTCCAACCACTG | |  |  |  |  |
| Zebrafish crfb13_cloning _F | | CGTCTGAAATCGGCCTGAAAAG | | cDNA cloning | | NM_001171592.1 | |
| Zebrafish crfb13_cloning _R | | ATCGGTGGTGCTAAAAACAAGGC | |  |  |  |  |
| Zebrafish crfb14_cloning _F | | TGAATGAATAATGTCCTGTTCAG | | cDNA cloning | | NM_001197202.2 | |
| Zebrafish crfb14_cloning _R | | ATGAACAGAGCAAAACAACCTC | |  |  |  |  |
| Zebrafish crfb16_cloning _F | | TTTGACCTGGACTGACAGAGATC | | cDNA cloning | | NM_001130975.1 | |
| Zebrafish crfb16_cloning _R | | TTGTTTCAAGCCTTTCTGAC | |  |  |  |  |
| Zebrafish crfb17_cloning _F | | ACAGATCTCAACATGGGATTTAC | | cDNA cloning | | NM_001135979.2 | |
| Zebrafish crfb17_cloning _R | | ACAGCATTTGATTCAAACAAAAGG | |  |  |  |  |
| gbcrfb5_cloning _F | | AGCTCGATGGGGACATGAAGTCGCTCCGCTG | | cDNA cloning | | LC752697 | |
| gbcrfb5_cloning _R | | TGCCTCTCTTCTCTGAGCTTCATCTGTGCTG | |  |  |  |  |
| gbstat6_Y677F_F | | TGATGGATTCCTGCCATTCAAACTAACA | | Construction of dominant  negative mutants | | LC752696 | |
| gbstat6_Y677F_R | | ATGGCAGGAATCCATCAGGATTAGGGGG | |  |  |  |  |
| gbstat6_Y677W_F | | TGATGGATGGCTGCCATTCAAACTAACA | | Construction of dominant  negative mutants | | LC752696 | |
| gbstat6_Y677W_R | | ATGGCAGCCATCCATCAGGATTAGGGGG | |  |  |  |  |
